# Phase contrast CMR in the descending aorta as a supportive reference for severe aortic regurgitation

**DOI:** 10.1038/s41598-025-31268-8

**Published:** 2025-12-24

**Authors:** Frida Truedsson, Sinsia A. Gao, Christian L. Polte, Odd Bech-Hanssen, Åse A. Johnsson, Kerstin M. Lagerstrand

**Affiliations:** 1 Department of Medical Radiation Sciences, Institute of Clinical Sciences, Sahlgrenska Academy at University of Gothenburg, Gothenburg, Sweden; 2 Department of Medical Physics and Biomedical Engineering, Region Halland, Halland Hospital, Halmstad, Sweden; 3 Institute of Medicine, Sahlgrenska Academy at University of Gothenburg, Gothenburg, Sweden; 4 Department of Clinical Physiology, Sahlgrenska University Hospital, Gothenburg, Sweden; 5 Department of Radiology, Region Västra Götaland, Sahlgrenska University Hospital, Gothenburg, Sweden; 6 Department of Radiology, Institute of Clinical Sciences, Sahlgrenska Academy at University of Gothenburg, Gothenburg, Sweden; 7 Department of Biomedical Engineering and Medical Physics, Region Västra Götaland, Sahlgrenska University Hospital, Gothenburg, Sweden; 8 MR-centre, Sahlgrenska University Hospital, Bruna stråket 13, Göteborg, 41345 Sweden

**Keywords:** Phase contrast cardiovascular magnetic resonance, Descending aorta, Aortic regurgitation, Regurgitation volume, Regurgitation fraction, Aortic regurgitation severity, Cardiology, Diseases, Medical research

## Abstract

Accurate grading of aortic regurgitation (AR) severity is crucial for treatment decisions. This study aimed to determine whether phase contrast cardiac magnetic resonance (PC-CMR) in the descending aorta can be used as a supporting reference for grading of the AR severity in patients with chronic AR. PC-CMR was performed both in the ascending and descending aorta of 191 AR-patients. Regurgitation thresholds in the descending aorta for hemodynamically significant AR [regurgitation volume (RVol)_DA_ and fraction (RF)_DA_] were determined in 41 well-controlled patients and then validated in a cohort surveyed for AR in our clinic. Myerson’s outcome-based thresholds (RVol > 42 mL, RF > 33%) served as reference. For comparison, holodiastolic flow reversal (HFR) was determined. Regurgitation measurements in the descending and ascending aorta were strongly correlated (RVol: *R* ≥ 0.92; RF: *R* ≥ 0.85; *p* < 0.001). Diagnostic performance of RVol_DA_>17 mL and RF_DA_>23% was high (sensitivity: 92%, 83%; specificity: 95%, 93%) and more patients with significant AR were identified using these PC-MRI based thresholds [RVol_DA_>17mL (13%) and RF_DA_>23% (15%)] than with HFR alone. PC-CMR in the descending aorta has the potential to reliably grade AR severity in patients with chronic AR and thereby may serve as a supportive reference in the decision-making regarding optimal timing of intervention.

**Supplementary Information:**

The online version contains supplementary material available at 10.1038/s41598-025-31268-8.

## Introduction

Aortic regurgitation (AR) occurs when the aortic valve fails to close properly, allowing blood to leak back into the left ventricle, potentially leading to irreversible heart damage over time. Patients with AR may need aortic valve replacement or repair, but the timing of intervention is a balancing act between too early with associated unnecessary surgical risk and too late leading to poorer prognosis with absence of recovery due to late intervention^1–3^. Grading the severity of AR helps in the decision-making of the optimal timing of the intervention^4^.

Two-dimensional echocardiography is currently the first-line diagnostic tool for assessment of AR severity^4–6^. Cardiovascular magnetic resonance (CMR), currently used for second-line diagnosis, can offer comprehensive evaluation^6,7^. AR can be assessed using through-plane phase-contrast (PC)-CMR measurements in the ascending aorta to directly quantify the regurgitated volume (RVol) and fraction (RF) through the leaking valve.

Generally, PC-CMR has been shown to assess the regurgitant flow accurately^6^. However, alternative imaging strategies are sometimes required to enhance diagnostic certainty^8,9^, since complex blood flow in the ascending aorta can affect the flow measurements^10^, and through-plane heart motion may underestimate regurgitant volume, particularly in cases of aortic dilation^11,12^. Patients with a mechanical aortic valve or those who have undergone transcatheter aortic valve implantation (TAVI) may also present diagnostic challenges^13^, particularly when complex vascular morphology restricts the ability to position the imaging plane freely^14^.

Indirect regurgitation references based on left ventricular volumes have been proposed as supporting reference, though they exhibit wide limits of agreement^6^. The diastolic flow reversal (DFR) velocity, measured in the proximal descending aorta, has been suggested as a predictor of severe AR^15^. Additionally, holodiastolic flow reversal (HFR), measured in the mid-descending thoracic aorta, has been shown to indicate severe AR^16,17^. Quantitative PC-CMR measurements in the descending aorta may potentially strengthen the diagnostic certainty of AR severity assessment.

Myerson et al. have proposed outcome-based PC-CMR thresholds in the ascending aorta^9^ that can identify patients with severe AR with future need for intervention^8,9,12^. The aim of this study was to develop similar thresholds for the descending aorta as supportive reference for AR severity in patients with chronic AR.

## Methods

### Study cohort and design

**Fig. 1:**
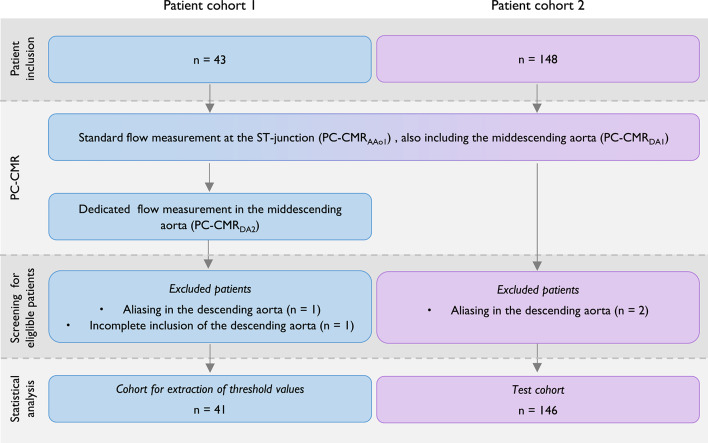
Flow diagram describing the patient inclusion of eligible patients.

The study included 191 patients with chronic AR, enrolled (1) in a carefully monitored design setting with well-controlled cohort and data collection, and (2) from clinical practice. Cohort 1 (*n* = 43, 24–80 years, 16% (7/43) females) was used to determine thresholds, and cohort 2 (*n* = 148, 20–80 years, 32% (47/148) females; Fig. 1) to validate their generalizability and diagnostic performance in a clinical setting. A subset of patients from cohort 1 was previously included in the study by Polte et al.^8^, where flow values from CMR were compared with those from echocardiography.

Figure 1 displays a flow diagram illustrating the inclusion of the patients. Inclusion criteria for cohort 1: Indication for intervention with severe chronic AR or follow-up with moderate chronic AR according to echocardiography. Patients in cohort 2 had clinical indications, i.e. known or suspected AR, and were included from the clinical flow between 2012 and 2018. Exclusion criteria were: aortic prosthetic valve, moderate regurgitation in any other valve, intracardiac shunt, any other form of cardiac disease, and irregular heart rhythm.

The study was conducted according to the Declaration of Helsinki. The Regional Ethics Review Board gave ethical approval for the study (2011-02-28, 075 − 11; 2018-06-20, 395 − 18). Written informed consent was obtained from all participants in cohort 1.

### Cardiovascular magnetic resonance

CMR was performed on a 1.5 T CMR scanner (Achieva, Philips Healthcare, Best, The Netherlands) using the five-channel phased-array cardiac coil. After standardized patient-specific planning, a series of cine-images were acquired in accordance with current guidelines. First, whole-heart images were acquired in the short-axis view without gap from the atrioventricular ring to the apex, followed by long-axis projections. All cine-images were acquired using balanced steady-state free precession sequences (TR = 3.4ms, TE = 1.7ms, and flip angle = 60°) with retrospective ECG gating (30 phases/cardiac cycle) and parallel imaging (acc factor = 2) during expiratory breath-hold. The in-plane resolution was typically 1.7 × 1.7mm^2^ with a slice thickness of 8 mm.

**Fig. 2:**
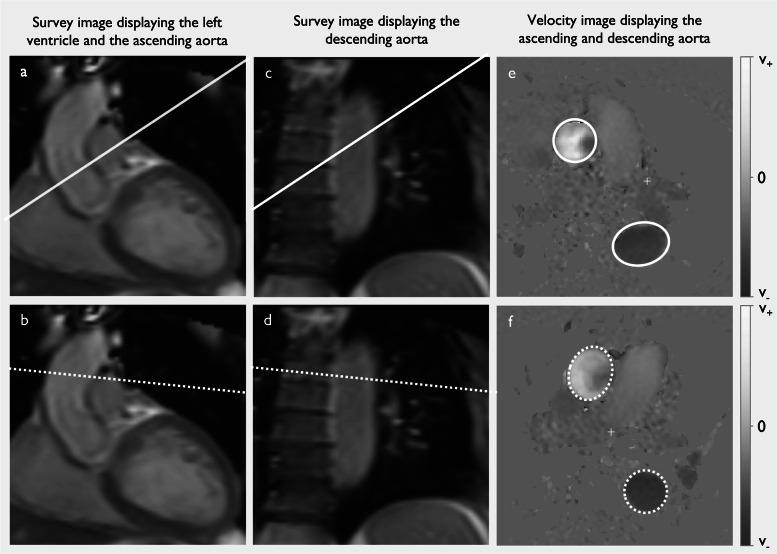
Illustration of the positioning of image planes for flow measurements: a, b) ascending aorta; c, d) descending aorta; and e, f) velocity images. In standard flow measurement (a, image plane 1, white solid line), the plane is positioned orthogonal to ascending aortic blood flow (PC-CMR_Ao1_), where the ascending aorta appears circular with minimal angulation errors (e). Standard flow measurement includes the descending aorta (c), allowing evaluation of blood flow at that position (PC-CMR_DA1_). However, the image plane is often angulated relative to descending aortic blood flow, causing the vessel lumen to appear elliptical (e) and introducing potential angulation errors in flow determination A dedicated measurement in the descending aorta (d, image plane 2, dotted white line) reduces errors by positioning the plane orthogonal to descending aortic blood flow (PC-CMR_DA2_), making the descending aorta appear circular (f).

PC-CMR with retrospective ECG gating were performed in accordance with current guidelines at the level of the sinotubular (ST)-junction during expiratory breath-hold (slice thickness = 8 mm, voxel size = 2.5 × 2.5mm^2^, TR = 4.8ms, TE = 2.9ms, BW = 477.8 Hz/pixel, flip angle = 12°, phases/cardiac cycle = 40, acc factor = 2, turbo field echo factor = 4, turbo field echo shots = 13 and number of averages = 1, field-of-view = 320 × 320mm^2^, acquisition matrix = 128 × 128, VENC = 180 cm/s (range:90–480 cm/s) (Fig. 2). The image plane was planned orthogonal to blood flow using the flow-induced signal void in cine-images, and velocity encoding for PC-CMR was optimized to the systolic blood flow. The VENC was individually adjusted to ensure that peak flow velocities were lower than the VENC but did not differ by more than 20%.

To avoid wraparound artifacts in the PC-CMR images, phase encoding was chosen in the narrowest anatomic direction. To optimize the temporal resolution, special care was taken to reduce the repetition time and the turbo factor. Background offset compensation was applied to reduce eddy current-induced velocity offsets using post-acquisition adaptive filtering, resulting in a velocity offset below the acceptance limit (0.6 cm/s)^18^.

All patients were examined with the same CMR protocol (Fig. 2). Cohort 1 was also imaged with a dedicated flow measurement in the descending aorta (PC-CMR_DA2_), where the image plane was positioned orthogonal to the direction of the descending aortic blood flow at the level of the pulmonary trunk (Fig. 2d)^14^. The PC-CMR acquisition was repeated twice in succession to assess reproducibility.

### Image analysis

Image analysis was performed using the Segment v1.9 R2046 research tool^19^, with the observer blinded to clinical data. The descending and ascending aorta were delineated on the PC-CMR magnitude image. The resulting segmentation masks were then copied onto the PC-CMR velocity image and propagated through all phases using a semi-automated tracking algorithm. Manual adjustment was performed if necessarily. From the calculated flow rate, regurgitation volume (RVol) and fraction (RF) in the ascending (RVol_AAo_, RF_AAo_) and descending aorta (RVol_DA_, RF_DA_) were determined. RVol and RF were calculated as the diastolic backward flow volume in mL and percentage of the positive flow volume. The reproducibility in these PC-CMR metrics between the ascending and descending aorta were compared. For comparison, presence of HFR was determined from the net flow rate curves, where presence of HFR was defined as flow reversal with a minimum flow of 10mL/s, persisting through the entire diastole^16^. Also, the DFR velocity was determined according to Hlubocká Z et al., where DFR velocity greater than 19.5 cm/s was used to predict severe AR^15^.

As a sub-analyses, the systolic backward flow volume was determined to evaluate the degree of complex blood flow in the ascending and descending aorta. Also, the distance from the aortic valve to the image plane in the descending aorta and the angulation of the image plane relative to the blood flow direction in the descending aorta were measured in the survey images to evaluate their possible influence on the results. Additionally, the aortic diameter in end-systole was measured and reported as normal (diameter < 40 mm) or dilated (≥ 40 mm)^20^.

### Determination of threshold values in the descending aorta

Thresholds for hemodynamic significant AR for the descending aorta (both for RVol_DA_ and RF_DA_) were determined using the Myerson’s outcome-based thresholds for the ascending aorta as reference (RVol > 42mL, RF > 33%^9^,. For that purpose, associations between these flow metrics in the ascending and descending aorta were estimated. In the estimations, the dedicated flow measurement in the descending aorta was used to minimize the influence of angle-related errors^14^.

### Statistical analysis

Statistical analyses were performed using MATLAB (R2018a, The MathWorks, Inc., Natick, Massachusetts, United States, 2018) and IBM SPSS Statistics 19 (IBM Corporation, Somers, New York). Unless otherwise stated, all values are expressed as the mean ± standard deviation (SD). A Wilcoxon signed-rank test was used for the comparison of dependent groups, and a Mann Whitney U-test was used for comparison of independent groups. Statistical differences with *p* < 0.05 were considered significant. To compare multiple groups, Friedman’s test was used. In cases where the null hypothesis was rejected, a post-hoc analysis of inter-group comparisons using the Wilcoxon signed-rank test was applied. The Bonferroni correction for multiple testing was used, with the null hypothesis rejected if *p* < 0.016. The degree of linear correlation was assessed by the Pearson correlation coefficient (R), where the spread of residuals was evaluated using the root mean square error (RMSE). In addition, the agreement between measurements was assessed using Bland–Altman analysis, where the mean difference and limits of agreement (± 1.96 SD) were calculated to evaluate systematic bias and the spread of differences. To determine the association between regurgitation estimates in the ascending and descending aorta and thereby enable estimation of threshold values for the descending aorta, a linear fit model was adapted to the PC-CMR data in cohort 1. Receiver operating characteristic (ROC) analysis was used as part of the validation process to evaluate the discriminatory power of the thresholds via the area under the curve (AUC). The diagnostic performance of the threshold values and the presence of HFR was described using sensitivity, specificity, as well as positive and negative likelihood ratios (PLR, NLR).

## Results

### Study cohorts

All patients were successfully examined. During the analysis stage, however, two individuals in cohort 1 were excluded due to velocity aliasing and incomplete inclusion of the descending aorta in the image (Table 1; Fig. 1). Likewise, two individuals in cohort 2 were excluded due to aliasing in the descending aorta.

**Table 1:** Demographics of included patients with chronic aortic regurgitation.

	All patients (*n* = 191)	Cohort 1 (*n* = 41)	Cohort 2 (*n* = 146)	*p*-value
Age (y)	49 ± 17	52 ± 15	48 ± 17	0.284
Sex, female (n(%))	53(28)	7(17)	46(32)	0.071
BSA (m^2^)	2 ± 0.2	2 ± 0.2	2 ± 0.2	0.375
Heart rate (beats/min)	66 ± 11	61 ± 9	67 ± 12	0.007
Systolic BP (mm Hg)	137 ± 20	136 ± 23	137 ± 19^a^	0.560
Diastolic BP (mmHg)	70 ± 15	68 ± 13	72 ± 15^a^	0.235
BAV (n(%))	75(40)	22(54)	53(36)	0.033
Concomitant AS (n(%))	16(9)	0	16(11)	0.030
LVEF (%)	55 ± 10	58 ± 7	55 ± 11	0.089
LVEDV (mL)	253 ± 86	309 ± 87	237 ± 80	< 0.001
LVESV (mL)	117 ± 57	132 ± 50	113 ± 58	0.016
LVSV (mL)	136 ± 46	177 ± 46	124 ± 39	< 0.001

Data presented as mean ± standard deviation (SD). Significance is presented as p-value. *AS*: aortic stenosis, *BAV*: bicuspid aortic valve, *BSA*: body surface area, *BP*: blood pressure, *EF*: ejection fraction, *LVEDV*: left ventricular end diastolic volume, *LVESV*: left ventricular end diastolic volume.

^a^ Data from 71 patients.

Table 2 show the findings for the final cohorts. RVol and RF were significantly larger in the ascending than in the descending aorta but no significant differences in these metrics were found between PC-CMR_DA1_ and PC-CMR_DA2_ for cohort 1 despite the fact that PC-CMR_DA2_ was found to differ in angulation from PC-CMR_DA1_ (8 ± 7°; range:0–30° vs. 33 ± 9°, range:15–62°, *p* < 0.001), and in distance from the aortic valve to the image plane in the descending aorta (16 ± 3 cm, range:11–25 cm vs. 15 ± 3 cm range:9–23 cm, *p* < 0.001). The ascending aorta was significantly larger than the descending aorta, and the degree of complex flow measured in terms of systolic backward flow volume was much smaller in the descending than in the ascending aorta (cohort 1:−2 ± 3 vs. −23 ± 18mL, *p* < 0.001; cohort 2:−3 ± 4 vs. −20 ± 15mL, *p* < 0.001, Supplementary Fig. S1). Also, no significant differences in the reproducibility of RVol and RF between the ascending and descending aorta were found for patients in cohort 1 with normal ascending aortas (reproducibility [RVol]:15 ± 16 vs. 13 ± 17%, *p* = 0.484, reproducibility[RF]:17 ± 17 vs. 16 ± 20%, *p* = 0.648). Higher reproducibility’s in the descending aorta than in the ascending aorta were found for patients in cohort 1 with dilated ascending aortas (reproducibility[RVol]:11 ± 14 vs. 24 ± 32% *p* = 0.055, reproducibility[RF]:13 ± 14 vs. 31 ± 40%, *p* = 0.026).

**Table 2:** Phase contrast magnetic resonance imaging findings for patients in cohort 1 (*n* = 41) and cohort 2 (*n* = 146).

	PC-CMR_AAo_	PC-CMR_DA1_	PC-CMR_DA2_	Friedman’s test *p*-value	Post-hoc analysis *p*-value			
					a	b	c	
*Cohort 1*								
Aortic diameter (mm)	38 ± 7	32 ± 6	28 ± 4	< 0.001	< 0.001	< 0.001	< 0.001	
RVol (mL)	61 ± 37	28 ± 23	29 ± 23	< 0.001	< 0.001	< 0.001	0.804	
RF (%)	38 ± 16	28 ± 18	28 ± 19	< 0.001	< 0.001	< 0.001	0.581	
*Cohort 2*								
Aortic diameter (mm)	37 ± 7	30 ± 6	-	-	< 0.001	-	-	
RVol (mL)	31 ± 23	12 ± 12	-	-	< 0.001	-	-	
RF (%)	26 ± 15	17 ± 14	-	-	< 0.001	-	-	

Data presented as mean ± standard deviation (SD). Significance is presented as p-value for comparisons: (a) PC-CMR_Aao_ vs. PC-CMR_DA1_, (b) PC-CMR_Aao_ vs. PC-CMR_DA2_, and (c) PC-CMR_DA1_ vs. PC-CMR_DA2_. *PC-CMR*: phase-contrast cardiac magnetic resonance, *PC-CMR*_*AAo*_ *and PC-CMR*_*DA1*_: PC-CMR in the ascending aorta and descending aorta (same image plane as for the ascending aorta*)*, *PC-CMR*_*DA2*_: dedicated PC-CMR in the descending aorta, *RF*: regurgitation fraction, *RVol*: regurgitation volume.

### Associations between the descending aorta and reference flow measurements

**Fig. 3:**
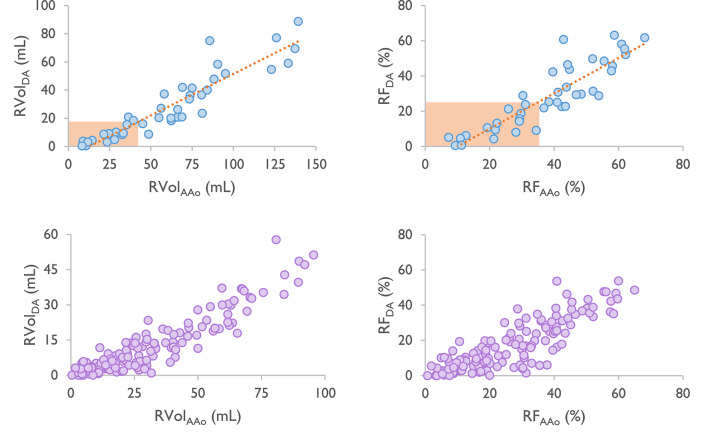
Upper panel: Correlation plots showing the association for RVol (left) and RF (right) between the ascending (PC-CMR_AAo1_) and descending aorta (PC-CMR_DA2_) in cohort 1. Linear equation [RVol]:$\:y=0.59x-7.43$, R-value[RVol]=0.93, linear equation[RF]:$\:y=1.01x-10.04$, R-value[RF] = 0.89. The orange box visualizes calculated threshold values for the descending aorta (17mL, 23%) based on Myerson’s threshold values for the ascending aorta (42mL, 33%)^9^. Lower panel: Correlation plots showing the association for RVol (left) and RF (right) between the ascending (PC-CMR_AAo1_) and descending aorta (PC-CMR_DA1_) in cohort 2.

For patients in both cohorts, a very strong association was found between the regurgitation metrics in the descending and ascending aorta (Fig. 3; Table 3). The Bland–Altman analyses further displayed this association as generally higher regurgitation values in the ascending aorta compared with the descending aorta, where the degree of the underestimation depended on the absolute RVol value: cohort 1, RVol 32.3 mL (−2.3–66.9), RF 9.4% (−7.8–26.6); cohort 2, RVol 18.3 mL (−6.9–43.4), RF 9.3% (−6.9–25.4) (Supplementary Fig. S2).

**Table 3:** Comparison of regurgitation volume and fraction between the ascending and descending aorta. The table presents pearson correlation coefficient (R-values), 95% confidence intervals (CI), statistically significant level (p-values), and root mean square errors (RMSE) from the correlation analysis in cohort 1 (*n* = 41) and cohort 2 (*n* = 146).

	*R*-value (95% CI)	*p*-value	RMSE
*Cohort 1*,* PC-CMR*_*AAo*_ *and* *PC-CMR*_*DA2*_			
RVol	0.93 (0.86–0.96)	< 0.001	9mL
RF	0.89 (0.79–0.94)	< 0.001	9%
*Cohort 2*,* PC-CMR*_*AAo*_ *and* *PC-CMR*_*DA1*_			
RVol	0.92 (0.89–0.94)	< 0.001	5mL
RF	0.85 (0.80–0.89)	< 0.001	7%

*PC-CMR*: phase-contrast cardiac magnetic resonance, *PC-CMR*_*AAo*_ *and PC-CMR*_*DA1*_: PC-CMR in the ascending aorta and descending aorta (same image plane as for the ascending aorta*)*, *RF*: regurgitation fraction, *RVol*: regurgitation volume.

### Thresholds and diagnostic performance

The ROC analyses showed that PC-CMR in the descending aorta could predict hemodynamic significant AR with strong discriminatory power (AUC[RVol] = 0.97, AUC[RF] = 0.96, Supplementary Fig. S3).

Using the linear equations from the correlation analysis between flow measurements in the ascending and descending aorta (Fig. 3, upper panel), threshold values for hemodynamic significance AR in the descending aorta were calculated as > 17mL for RVol_DA_ and > 23% for RF_DA_.

**Fig. 4:**
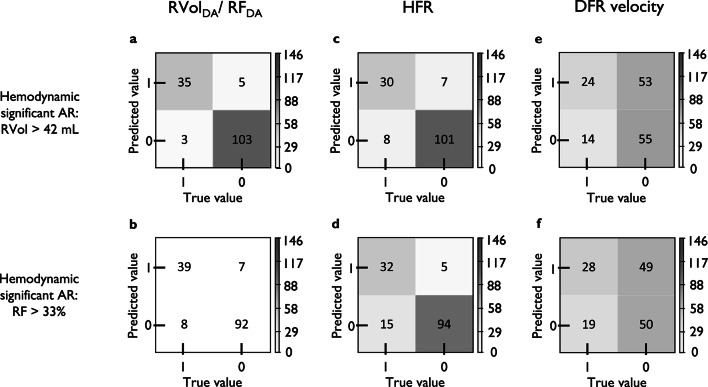
Confusion matrices from validation analysis of threshold values for (**a**) RVol_DA_ and (**b**) RF_DA_, as well as for HFR and DFR velocity with c, e) RVol_AAo_>42mL as reference^9^ and d, f) RF_AAo_>33% as reference^9^, in cohort 2 (Fig. 1). Each matrix shows four different combinations of true and predicted values: True Positive (1,1), True Negative (0,0), False Positive (0,1), and False Negative (1,0). The number in each quadrant refers to this description (number of patients). The image plane angulations for False Positive patients (0,1) were: RVol = 23 ± 5°, RF = 26 ± 13°, HFR[RVol] = 23 ± 8°, HFR[RF] = 20 ± 7°, DFR[RVol] = 34 ± 16°, DFR[RF] = 35 ± 16°. The image plane angulations for False Negative patients (1,0) were: RVol = 32 ± 3°, RF = 28 ± 10°, HFR[RVol] = 28 ± 5°, HFR[RF] = 28 ± 9°, DFR[RVol] = 27 ± 11°, DFR[RF] = 29 ± 10°. **Text tables**.

These thresholds could predict patients with hemodynamic significant AR in the clinical cohort with high sensitivity (RVol/RF = 92/83%) and specificity (95/93%; Figs. 1 and 4). The discriminatory ability was strong (PLR[RVol/RF]:20/12; NLR[RVol/RF]:0.08/0.18; Fig. 4). Compared to RVol_DA_ and RF_DA_, HFR showed lower sensitivity and NLR but equal specificity (Fig. 4; Table 4). The PLR was lower for HFR than RVol_DA_ but equal to RF_DA_ (Fig. 4; Table 3). RVol_DA_>17mL and RF_DA_>23% identified 13% (*n* = 5/38) and 15% (*n* = 7/47) more patients with hemodynamic significant AR compared to HFR (Fig. 4). The DFR velocity was found to predict patients with hemodynamic significant AR with low diagnostic performance, inferior to RVol_DA_, RF_DA_, and HFR in terms of sensitivity, specificity, PLR, and NLR.

**Table 4:** Diagnostic performance of threshold values that indicate hemodynamic significance aortic regurgitation.

	Threshold value	Sensitivity (95% CI)	Specificity (95% CI)	PLR (95% CI)	NLR (95% CI)
*Hemodynamic significant AR: RVol*_*AAo*_ *>42mL *^9^					
RVol_DA_	17mL	92 (79–98)	95 (89–99)	20.0 (8.5–47.4)	0.08 (0.03–0.24)
HFR	Flow reversal ≥ 10 ml/s during diastole	79 (63–90)	94 (87–98)	12.1 (5.6–26.5)	0.23 (0.12–0.42)
DFR velocity	> 19.5 cm/s	63 (46–78)	51 (41–61)	1.3 (1.0–1.7.0.7)	0.72 (0.49–1.08)
*Hemodynamic significant AR: RF*_*AAo*_ *>33% *^9^					
RF_DA_	23%	83 (69–92)	93 (86–97)	11.7 (5.8–23.8)	0.18 (0.10–0.33)
HFR	Flow reversal ≥ 10 ml/s during diastole	68 (53–81)	95 (89–98)	13.5 (5.9–31)	0.34 (0.22–0.51)
DFR velocity	> 19.5 cm/s	60 (44–74)	51 (40–61)	1.2 (0.9–1.6)	0.80 (0.56–1.14)

*AR*: aortic regurgitation, *DFR*: diastolic flow reversal, *HFR*: holodiastolic flow reversal, *NLR*: negative likelihood ratio, *PLR*: positive likelihood ratio, *RF*: regurgitation fraction, *RF*_*Aao*_: RF determined in the ascending aorta, *RF*_*DA*_: RF determined in the descending aorta, *RVol*: regurgitation volume, *RVol*_*Aao*_: RVol determined in the ascending aorta, *RVol*_*DA*_: RVol determined in the descending aorta.

## Discussion

This study identified strong correlations between RVol and RF in the ascending and descending aorta in patients with chronic AR, offering objective PC-CMR thresholds in the descending aorta that effectively distinguished hemodynamic significant AR from non-significant cases. Notable, the thresholds showed a high level of diagnostic accuracy in patients from our clinical practice. Further validations in larger multicenter studies are warranted to establish PC-CMR in the descending aorta as a supportive reference for severe AR.

Accurate grading of the AR severity is important for the timing of the intervention. Previous studies have shown that in some patients, AR may be underestimated with standard PC-CMR due to through-plane heart motion^12^. Also, complex flow and inhomogeneities near metallic aortic prosthetic valves may introduce errors in these measurements^10,13^. Hence, when diagnostic variability in results arises due to e.g. factors such as patient anatomy, imaging artifacts, or technical limitations, further investigations or alternative diagnostic methods are encouraged to clarify this uncertainty. For example, the AR severity assessment can be extended to other anatomical locations, like the descending aorta, to cross-check results and resolve the incongruence.

Here, we present findings that provide scientific evidence that PC-CMR in the descending aorta can serve as a reliable complement to standard PC-CMR and enhance the AR severity assessment. The measurement may even provide an alternative diagnostic reference for patients where standard PC-CMR is limited, such as patients with congenital heart disease and abnormal ascending aorta morphologies^14^. Table 5 presents a proposed clinical decision algorithm integrating findings with evidence-based guidelines.

**Table 5:** When to use ascending vs. descending aorta (AAo vs. DA) PC-CMR based on clinical context. Proposed clinical decision algorithm, integrating evidence-based guidelines and findings from the present study.

Clinical context	Recommendation
Normal AAo	AAo is primary, DA for comparison
Dilated AAo (associated with complex flow)	AAo and DA; consider DA if discrepancies occur
Poor image quality in AAo (artifacts or turbulence)	AAo and DA; consider DA if discrepancies occur
Abnormal aortic morphology (congenital heart disease)	AAo and DA; consider DA if discrepancies occur

Especially patients with TAVI may benefit from PC-CMR based thresholds in the descending aorta. Advancements in medical technology have increased the use of this minimally invasive intervention, making it possible to treat high risk patients who were previously ineligible for surgery. However, one of the concomitant complications is paravalvular leakage and in such cases, the assessment of the severity is crucial, but known to be difficult using echocardiography^21^. Also, standard PC-CMR in the ascending aorta near the stent, where the valve is positioned, poses a challenge since the stent is made of metal and thereby causes errors in the flow quantification^13^.

Reimold et al. have previously evaluated the possibility to assess AR severity using PC-CMR in the descending aorta^22^, but found only a weak correlation between the ascending and descending measurements. They attributed this finding to the normal variation of blood distribution in the head, arms, body, and lower extremities between individuals, but the lower correlation may have been attributed to the poorer technical performance of the PC-CMR measurement at that time as well as the small number of individuals (*n* = 16).

It is known that the PC-CMR based RVol and RF estimates are influenced by the position of the image plane^23^. The underestimation may occur due to physiological and hemodynamic factors such as the Windkessel effect and the branching of the blood flow to the head, neck, and arms^24,25^. Present findings seemed to demonstrate this effect, resulting in systematically lower values in the descending aorta. Also, the strong and significant correlation between the ascending and descending aorta indicates that the variation between individuals is both consistent and small, thus, does not impair the detection of hemodynamic significant AR in the descending aorta.

The included cohorts were similar with respect to age, sex distribution, body size, and blood pressure, but differed in heart rate. The observed difference in heart rate is unlikely to have affected the comparison of volumetric flow estimates (RVol and RF) between the groups. In PC-MRI, heart rate primarily influences peak velocity measurements, whereas volumetric flow is generally robust to heart rate variations, provided that gating and temporal resolution are adequate. The thresholds further demonstrated strong diagnostic performance, supporting the utility of the study.

In this study, we chosen to adopt the outcome-based regurgitation volume and fraction thresholds proposed by Myerson et al.^9^ as outlined in the Recommendations for Noninvasive Evaluation of Native^6^. The Meyerson approach integrates clinical outcomes and imaging data to assess the severity of regurgitation in a way that reflects its actual impact on the patient. As such, it may correlate more closely with clinically significant outcomes such as symptoms, left ventricular remodeling, and the progression of heart failure, providing a more functional and prognostic view of regurgitation severity than current guideline recommendations^4,5^. Research further suggests that the current Guidelines, which are based on echocardiography, presents slightly to high PC-CMR based thresholds and should be revised downward towards the Myerson’s outcome-based thresholds^8,9^. Regardless, it is important to emphasize that regurgitation thresholds should not be seen as rigid. Individual variability and the advanced capabilities of modern imaging techniques may contribute to differences in measured values. Strict adherence to thresholds can lead to over-treatment in patients with regurgitation values near the threshold but no symptoms or adverse remodeling. Conversely, it may under-treat patients who fall below the thresholds but have clinically significant impacts.

Another imaging reference for assessment of AR severity is the indirect CMR method based on left ventricular volumes^6^. However, this method has shown wide limits of agreement^8,26^. Also, HFR, has been suggested for the evaluation of AR^16^. Present findings show, in line by the work of Bolan et al.^9^, that presence of HFR can effectively predict severe AR with high sensitivity and specificity. We also assessed the presence or absence of HFR, allowing for a comparison with threshold values for RVol_DA_ and RF_DA_. Notably, our findings revealed a slightly larger number of patients with hemodynamic significant AR using the PC-CMR based thresholds compared with presence of HFR.

In contrast to Hlubocká Z et al., who reported that the DFR velocity could predict severe AR with high diagnostic performance^7^, we found that the diagnostic performance for DFR velocity was low. Hlubocká Z et al. determined the DFR velocity using Doppler echocardiography, while we used PC-CMR. The intrinsically low time and spatial resolution of the acquisition most probably limited the diagnostic performance of DFR using PC-CMR. While it could be hypothesized that the lower velocity-to-noise in the descending aorta may have influenced the precision of the DFR velocity, a study has shown that the velocity-to-noise has no significant impact on averaged velocity estimates, such as blood volume and mean flow velocities, in large vessels^27^.

Present study was conducted using 2D PC-CMR, but the findings are directly applicable to volume-based PC-CMR; a technique increasingly used in clinical practice. This “4D flow” technique allows for multiple flow measurements to be obtained from a single data acquisition. Additionally, it enables retrospective quantification of blood flow at any position and angulation of the image plane. Thus, 4D flow is an appealing alternative to conventional 2D PC-CMR for evaluating AR severity, as it allows optimized flow measurements in both the ascending and descending aorta to be derived from the same scan.

This study had some limitations. It was designed as a single study only which may limit the strength of the conclusions. The clinical cohort, i.e. cohort 2, included patients with both non-severe and severe AR and was considered a representative clinical cohort for identification of severe chronic AR and therefore suitable for validation of the determined threshold values. However, the patients in this cohort generally had lower RVol and RF than those in cohort 1 and the number of patients with severe AR was smaller than those with non-severe AR. While this may have impacted diagnostic performance, the strong sensitivity and specificity of the threshold values indicate otherwise. A potential limitation in the PC-CMR measurements of the clinical cohort may have been the angulation of the image plane in the descending aorta. In small vessels, image plane angulation affects blood flow accuracy due to partial-volume effects^14^, while the reduced velocity from angulation is offset by increased area in larger vessels. This explains why no difference was observed between dedicated flow measurements in the descending aorta (orthogonal plane) and intrinsic measurements from the ascending aorta (angulated plane). Further, while our study focused on volumetric flow measurements in large vessels due to limitations at the ascending aorta, the underlying etiology of AR, e.g. whether root or annular dilation or leaflet prolapse, may alter jet direction and flow characteristics. Finally, the findings of this study apply primarily to patients with chronic AR in sinus rhythm. The applicability to acute AR or to patients with irregular rhythms warrants further investigation.

To conclude, this study shows that PC-CMR in the descending aorta has the potential to reliably assess the AR severity in patients with chronic AR using developed regurgitation volume and fraction thresholds. The alternative diagnostic strategy may prove to be useful in the decision-making regarding optimal timing of intervention and support the diagnosis of hemodynamic significant AR. However, further validations are encouraged.

## Supplementary Information

Below is the link to the electronic supplementary material.

Supplementary Material 1

Supplementary Material 2

Supplementary Material 3

Supplementary Material 4

Supplementary Material 5

Supplementary Material 6

Supplementary Material 7

Supplementary Material 8

## Data Availability

The data is available from the corresponding author on reasonably request.
