# Supplementary material for: Phase contrast CMR in the descending aorta as a supportive reference for severe aortic regurgitation
Source: Sci Rep. 2025 Dec 24;15:44662. doi: 10.1038/s41598-025-31268-8 (PMC12749755; doi:10.1038/s41598-025-31268-8)
Supplement: Supplementary file 1 — Supplementary Material 1 [file 41598_2025_31268_MOESM1_ESM.docx]

**Supplementary infomation**

*Fig. S1.* Net flow rate curves (solid line) and negative flow rate curves (dashed line) obtained in the a,b) ascending and c,d) descending aorta in a,c) a patient with a normal ascending aorta (aortic diameter=33mm) and b,d) a patient with a dilated ascending aorta (aortic diameter=52mm)

*Fig. S2.* Upper panel: Bland-Altman plots, displaying the agreement for RVol (left) and RF (right) between the ascending (PC-CMR_AAo1_) and descending aorta (PC-CMR_DAo2_) in cohort 1. Lower panel: Bland-Altman plots, displaying the agreement for RVol (left) and RF (right) between the ascending (PC-CMR_AAo1_) and descending aorta (PC-CMR_DAo1_) in cohort 2

*Fig. S3.* Resultant receiver operating characteristic (ROC) curves for predicting hemodynamic significant aortic regurgitation in the descending aorta in cohort 1, for a) RVol_DA_ and b) RF_DA_. Orange dots indicate the calculated threshold values (17mL, 23%) for the descending aorta (Figure S1, upper panel)
