# Supplementary figures and images for: Phase contrast CMR in the descending aorta as a supportive reference for severe aortic regurgitation
Source: Sci Rep. 2025 Dec 24;15:44662. doi: 10.1038/s41598-025-31268-8 (PMC12749755; doi:10.1038/s41598-025-31268-8)

## Patient cohort 1

## Patient cohort 2

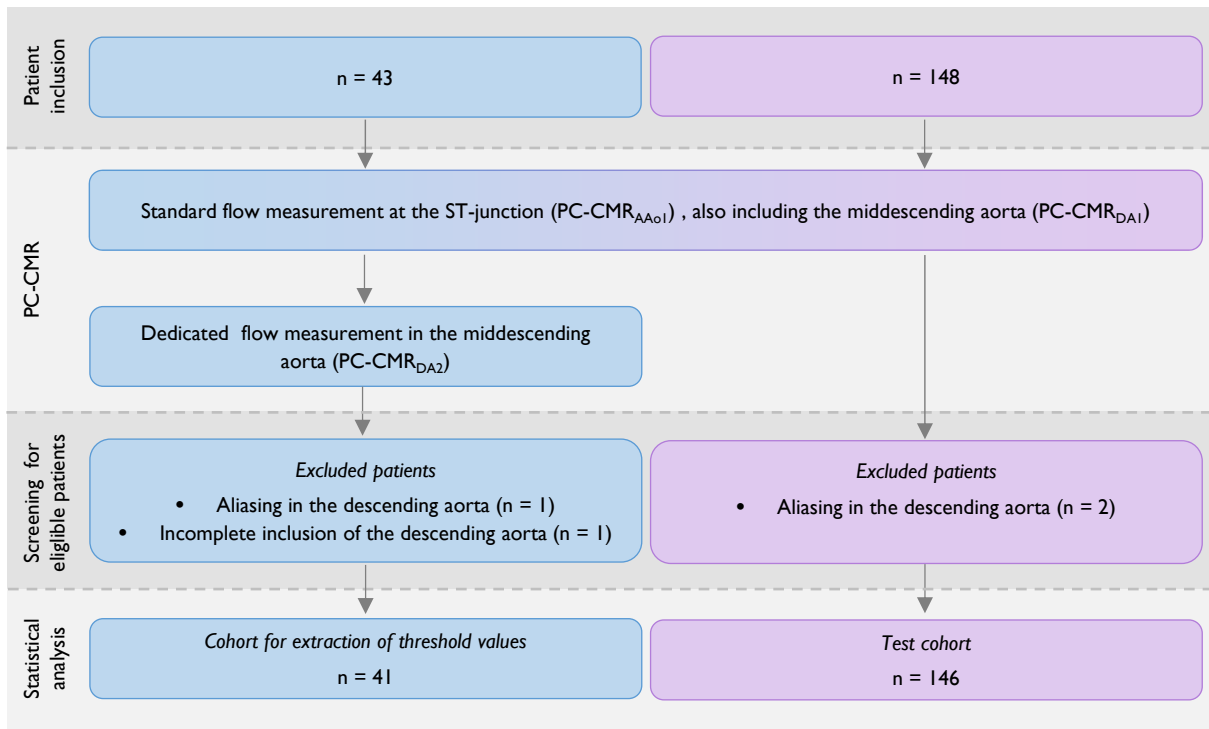

Supplement: Supplementary file 2 — Supplementary Material 2 [file 41598_2025_31268_MOESM2_ESM.pdf]

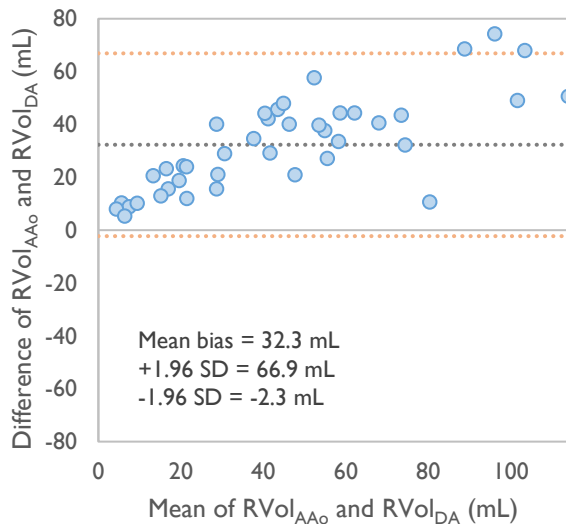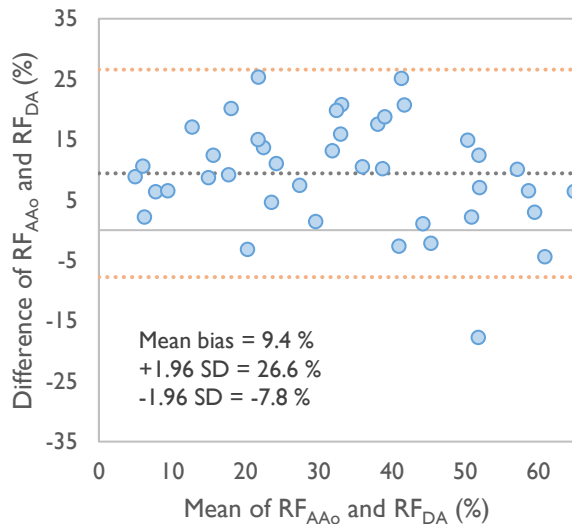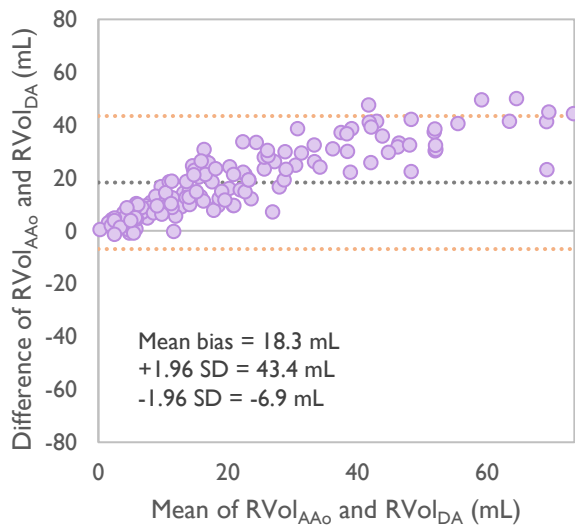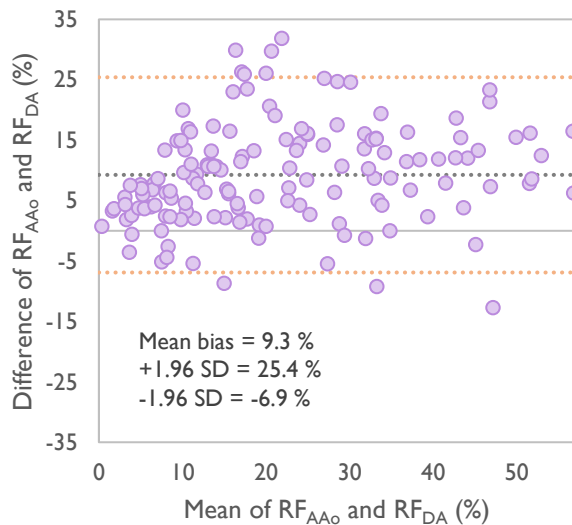

Supplement: Supplementary file 4 — Supplementary Material 4 [file 41598_2025_31268_MOESM4_ESM.pdf]

**a**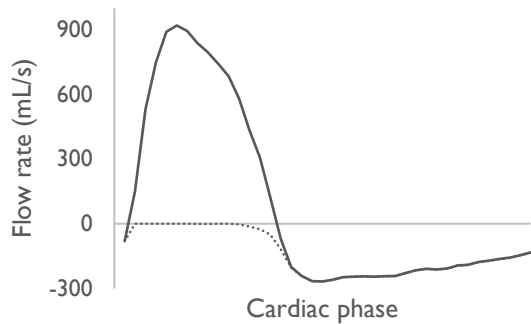**c**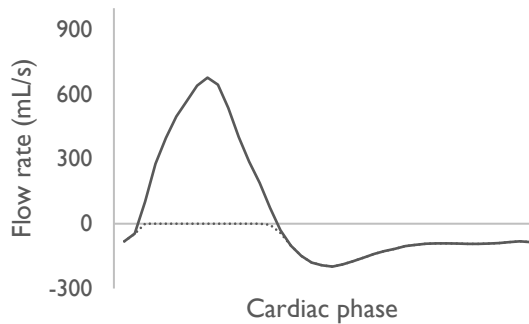**b**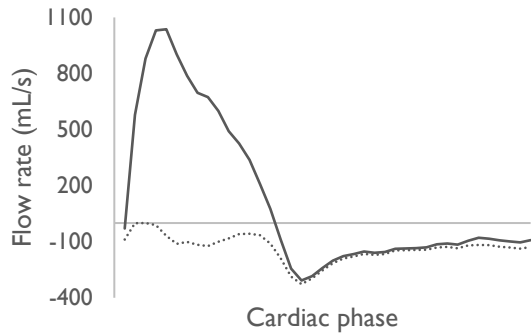**d**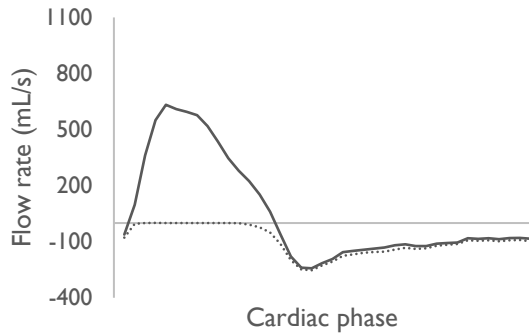

Supplement: Supplementary file 6 — Supplementary Material 6 [file 41598_2025_31268_MOESM6_ESM.pdf]

**a****RVol<sub>DA</sub>**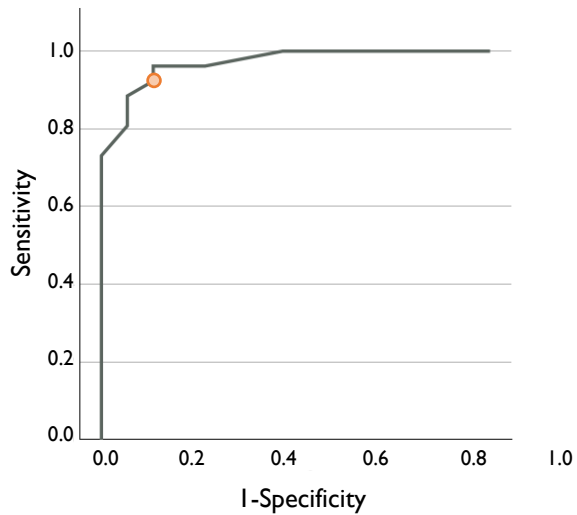**b****RF<sub>DA</sub>**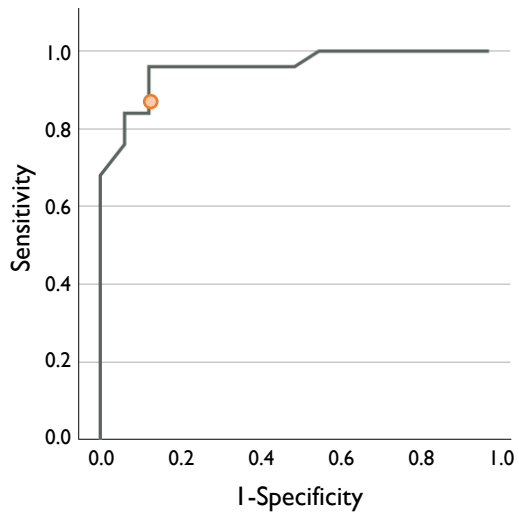

Supplement: Supplementary file 7 — Supplementary Material 7 [file 41598_2025_31268_MOESM7_ESM.pdf]

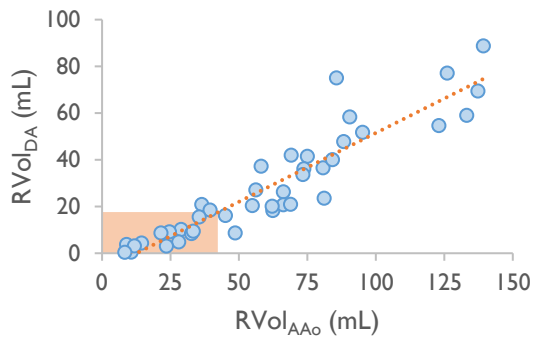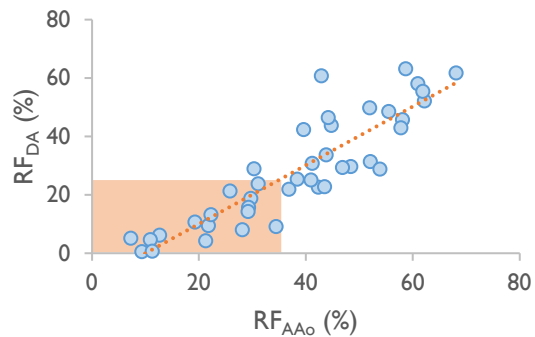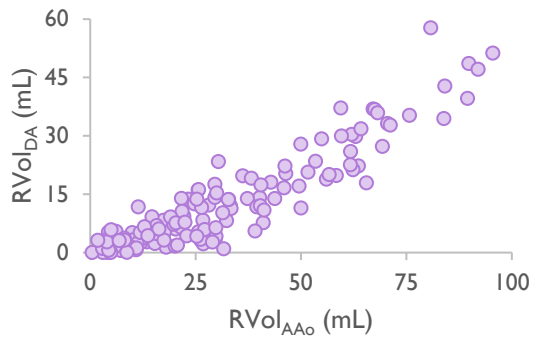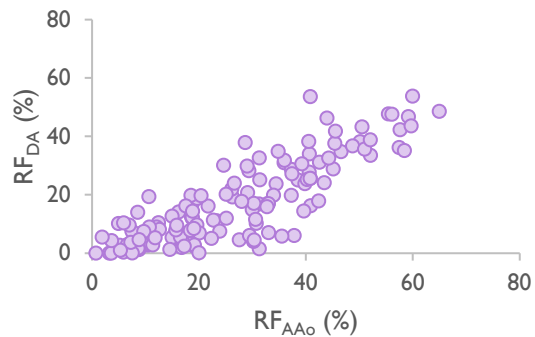

Supplement: Supplementary file 8 — Supplementary Material 8 [file 41598_2025_31268_MOESM8_ESM.pdf]
