# Supplementary material for: Phase contrast CMR in the descending aorta as a supportive reference for severe aortic regurgitation
Source: Sci Rep. 2025 Dec 24;15:44662. doi: 10.1038/s41598-025-31268-8 (PMC12749755; doi:10.1038/s41598-025-31268-8)
Supplement: Supplementary file 3 — Supplementary Material 3 [file 41598_2025_31268_MOESM3_ESM.pdf]

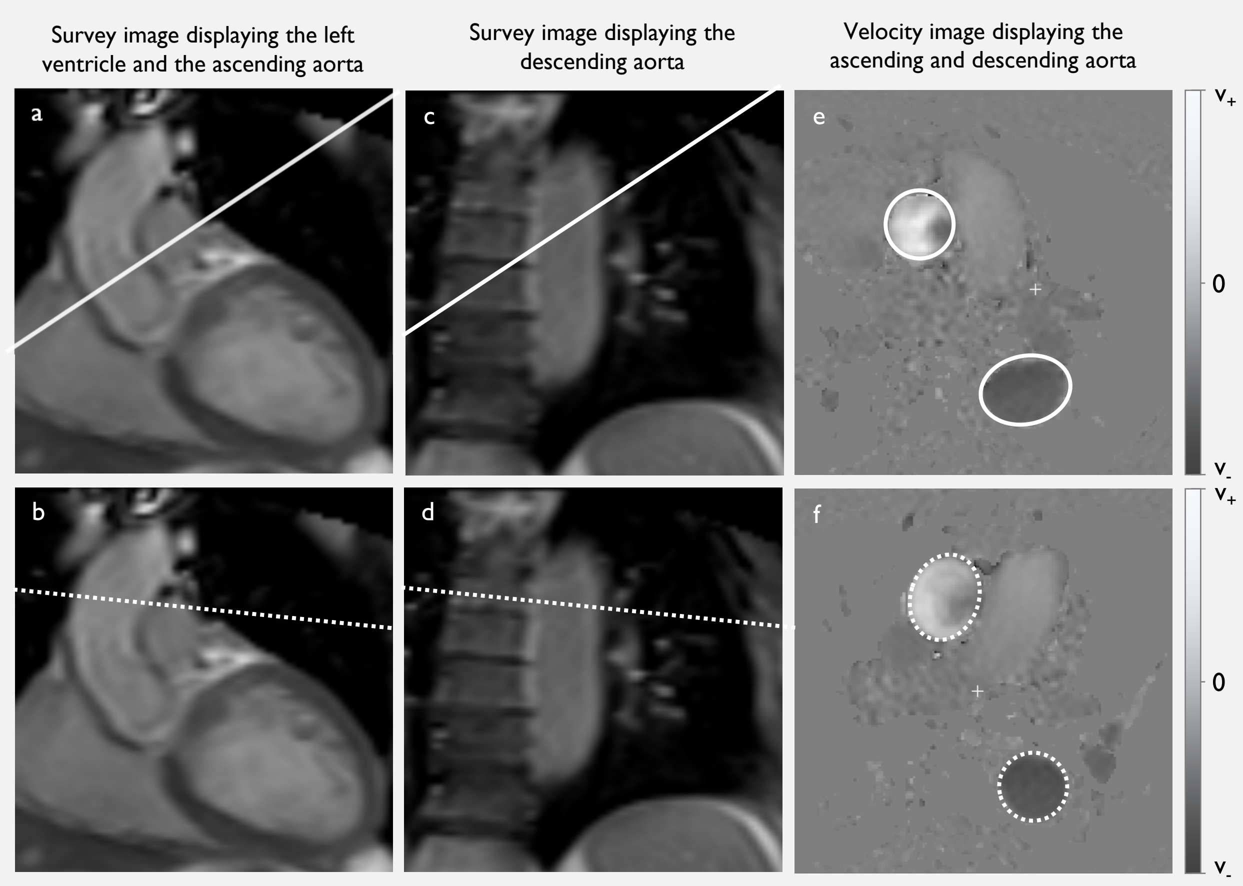
Survey image displaying the left ventricle and the ascending aorta
Survey image displaying the descending aorta
Velocity image displaying the ascending and descending aorta
a
b
c
d
e
f
v+
0
v-
v+
0
v-
