# Supplementary material for: Phase contrast CMR in the descending aorta as a supportive reference for severe aortic regurgitation
Source: Sci Rep. 2025 Dec 24;15:44662. doi: 10.1038/s41598-025-31268-8 (PMC12749755; doi:10.1038/s41598-025-31268-8)
Supplement: Supplementary file 5 — Supplementary Material 5 [file 41598_2025_31268_MOESM5_ESM.pdf]

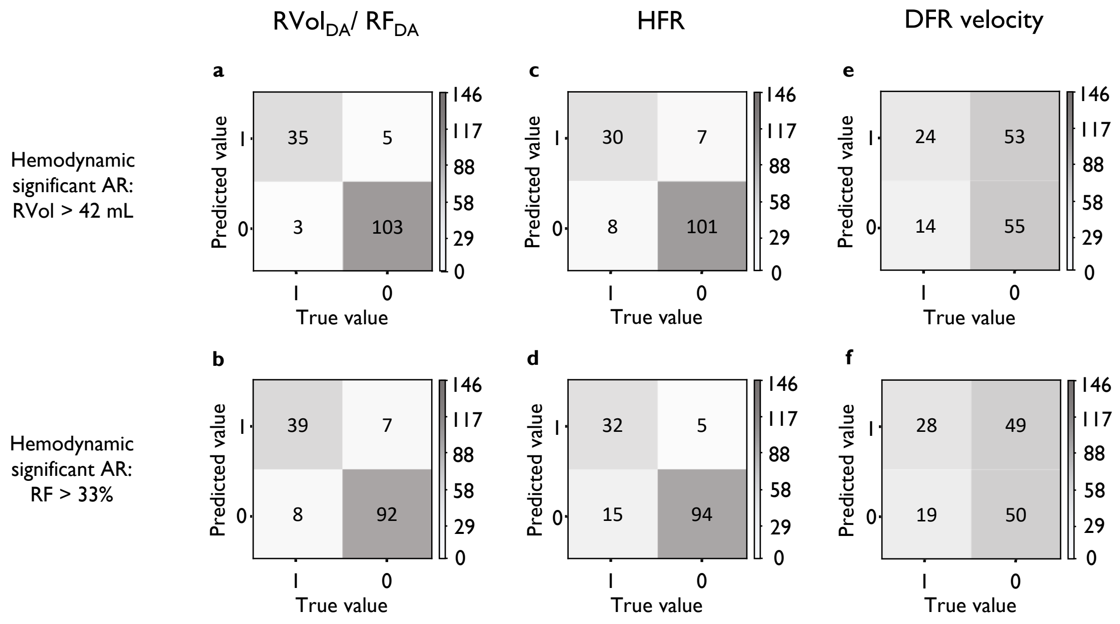

$RVol_{DA}$/ RF_{DA}
HFR
DFR velocity
Hemodynamic significant AR: RVol > 42 mL
Hemodynamic significant AR: RF > 33%
a
35
5
3
103
c
30
7
8
101
e
24
53
14
55
b
39
7
8
92
d
32
5
15
94
f
28
49
19
50
Predicted value
True value
1
0
146
117
88
58
29
0
